# Supplementary material for: Population Genetic Structure Is Unrelated to Shell Shape, Thickness and Organic Content in European Populations of the Soft-Shell Clam Mya Arenaria
Source: Genes (Basel). 2020 Mar 11;11(3):298. doi: 10.3390/genes11030298 (PMC7140805; doi:10.3390/genes11030298)
Supplement: Supplementary file 1 [file genes-11-00298-s001.zip › Supplementary_Material/Supplementary_Material_captions.docx]

**Supplementary Materials:**

**Supplementary Figure S1**: Scanning Electron Microscope (SEM) images of *M. arenaria* shell microstructure. Shell cross-sections were progressively polished (up to 1μm), ultrasonically cleaned and air-dried prior to mounting and sputter coating (Emitech K550X). Images were taken using a Quanta-650F SEM (Department of Earth Sciences, University of Cambridge, UK). The microstructural nomenclature of Bieler et al. [45] has been used. Scale bars are shown on the bottom right of each image. (a) the Periostracum, indicated by white arrows (b) the outer granular prismatic layer, (c) the middle crossed-lamellar layer; and (d) the inner complex crossed-lamellar layer (comprising a sequence of crossed-lamellar and prismatic layers shown by white arrows).

**Supplementary Figure S2:** Results of the calibration procedure used for elliptic Fourier analysis (EFA) of shell outlines. Cumulative harmonic Fourier power is shown separately for (a) lateral and (b) anterior shell views. The power is proportional to the harmonic amplitude and can be considered a measure of shape information [63]. We evaluated the appropriate number of harmonics to retain so that their cumulative power captured 99% of the total Fourier power. Average shell shapes reconstructed for different numbers of harmonics (1, 3, 5, 7 and 9) are shown.

**Supplementary Figure 3**: Example outcomes of Thermal Gravimetric Analysis (TGA, green line) and Derivative Thermogravimetry (DTG, blue line). The TGA curve represents weight changes with increasing treatment temperature for the granular prismatic layer of *Mya arenaria*. Four regions of weight loss with increasing temperature are highlighted: (i) the evaporation of physically adsorbed water at 30‒150 °C; (ii) the degradation of extra-crystalline organic matrix at 150-400 °C; (iii) the release of intra-crystalline organics at 400-550 °C; and (iv) the rapid decomposition of calcium carbonate (CaCO_3_) into calcium oxide (CaO) and carbon dioxide (CO_2_) starting at ~550 °C. The DTG line represents the derivative of the thermal curve and shows the rate of weight loss during heating. The peak indicates the temperature at which the organic mass loss was fastest.

**Supplementary Figure S4:** Contributions of PCs toward variation in shell shape for increasing PC values: mean -3 SD (blue), mean (black), and mean + 3 SD (red). PC1 contributed mainly towards variation in shell roundness and depth. By contrast, PC2 described variation in shell roundness and in the symmetry of the anterior view profile. PC3 contributed towards minor variation in the lateral view profile.

**Supplementary Figure S5**: Contribution of the first four shape variables (PCs) to shape variation. Average shell shapes for the lateral and anterior view are shown for increasing values along each PC (Mean - 3SD, Mean, Mean + 3SD) and shapes at the extremes of each variable are compared (Mean ±3 SD).

**Supplementary Figure S6**: Patterns of shell shape variations in European *M. arenaria* samples. (a) Mean shell shapes for each collection site. (b) Differences between mean shapes at the extremes of the morphospace represented through (i) iso-deformation lines (bottom), representing the outline regions subjected to different degrees of change (blue: low deformation; red: strong deformation), and (ii) deformation grids (top), depicting the bindings required to pass from an extreme (PLY) to another (SAN).

**Supplementary Table 1:** Details of the 12 microsatellite loci used in this study together with their annealing temperatures and polymorphism characteristics in 247 soft-shell clams sampled from nine different populations.

**Supplementary Table 2:** Alternative models of shell shape and shell layer thickness for optimal fixed and variance structures (shown in bold). Degrees of freedom (*k*), log likelihood estimation, corrected AIC values, and likelihood estimation methods are reported for each model.

**Supplementary Table 3:** Pairwise *F*_ST_ values (below diagonal) and corresponding *p*-values (above diagonal) calculated from 11 microsatellites. Bold *p*-values were significant only before table-wide FDR correction, while bold and underlined *p*-values also remained significant after FDR correction.

**Supplementary Table S4:** Summary of the results of the GLMM of whole shell thickness. Estimated statistics bootstrapped 95% CIs for regression parameters are reported for the modelled relationships between shell thickness, latitude, shell length and genetic variables. Regression parameters were considered statistically significant and highlighted in bold when the bootstrapped 95% CI did not overlap zero.
