# Supplementary figures and images for: Population Genetic Structure Is Unrelated to Shell Shape, Thickness and Organic Content in European Populations of the Soft-Shell Clam Mya Arenaria
Source: Genes (Basel). 2020 Mar 11;11(3):298. doi: 10.3390/genes11030298 (PMC7140805; doi:10.3390/genes11030298)

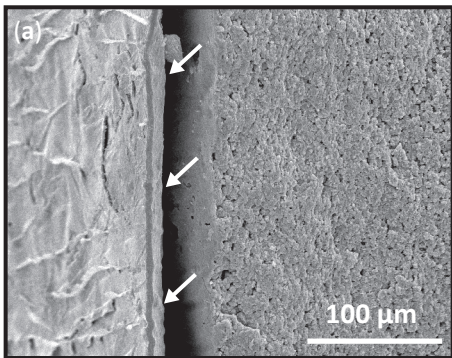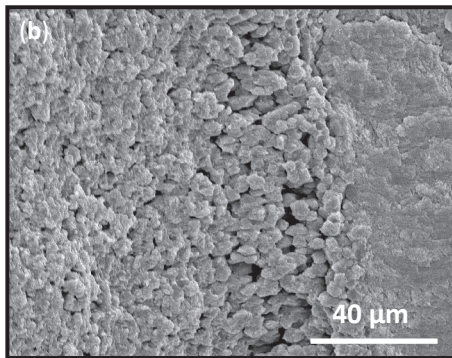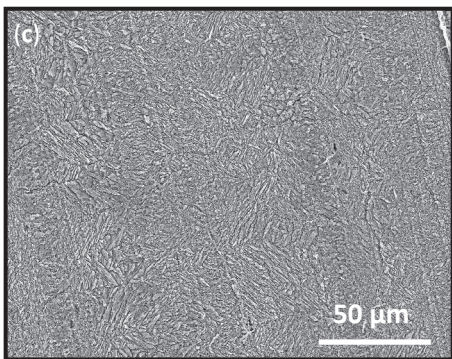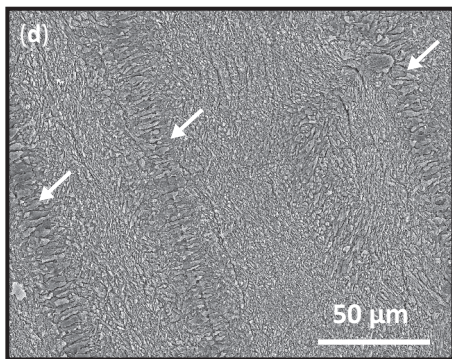

Supplement: Supplementary file 1 [file genes-11-00298-s001.zip › Supplementary_Material/SF_1.pdf]

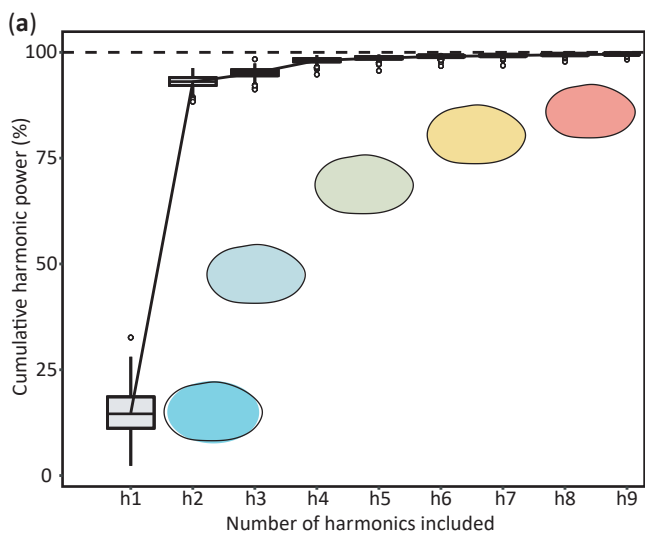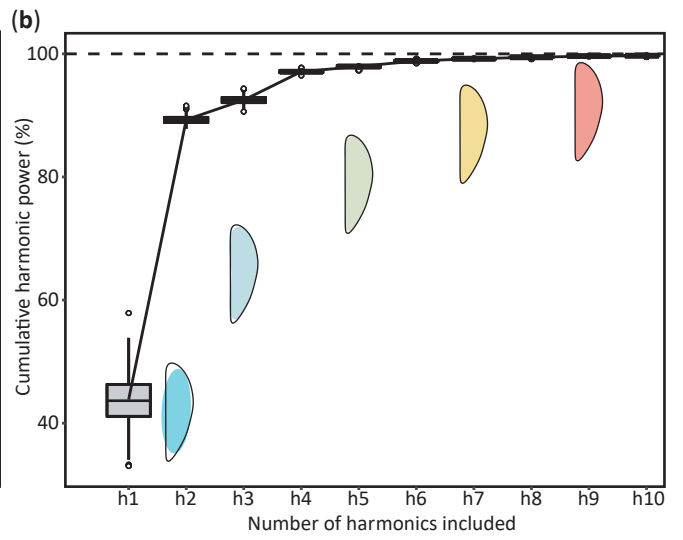

Supplement: Supplementary file 1 [file genes-11-00298-s001.zip › Supplementary_Material/SF_2.pdf]

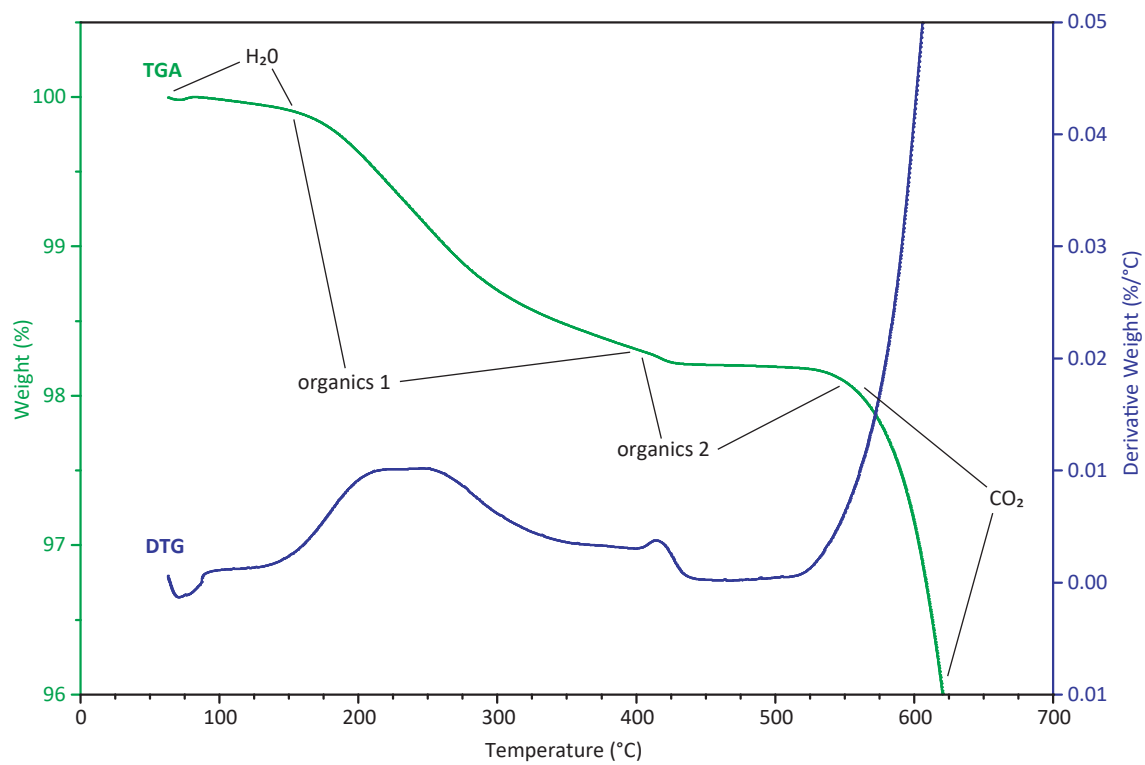

Supplement: Supplementary file 1 [file genes-11-00298-s001.zip › Supplementary_Material/SF_3.pdf]

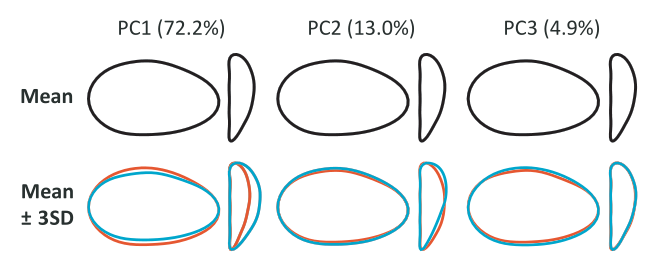

Supplement: Supplementary file 1 [file genes-11-00298-s001.zip › Supplementary_Material/SF_4.tiff]

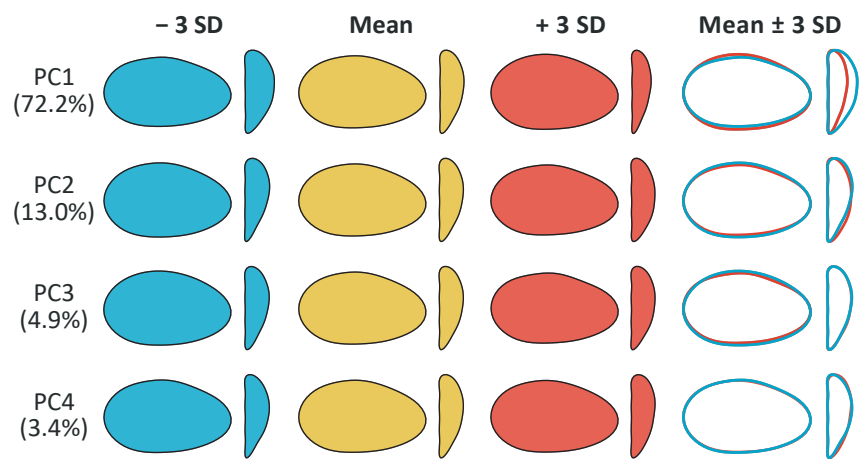

Supplement: Supplementary file 1 [file genes-11-00298-s001.zip › Supplementary_Material/SF_5.pdf]

(a)

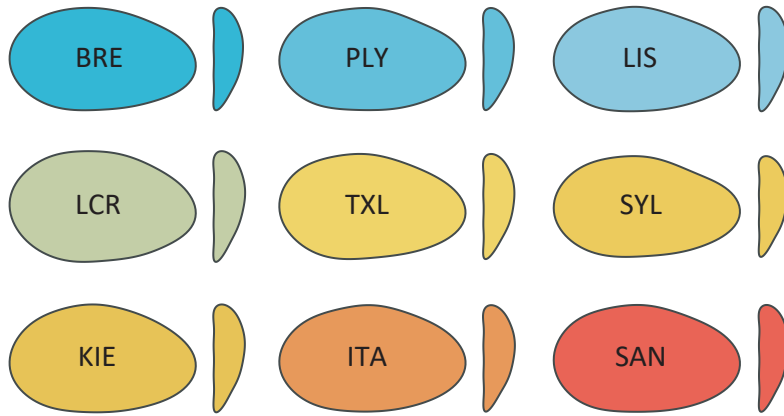

(b)

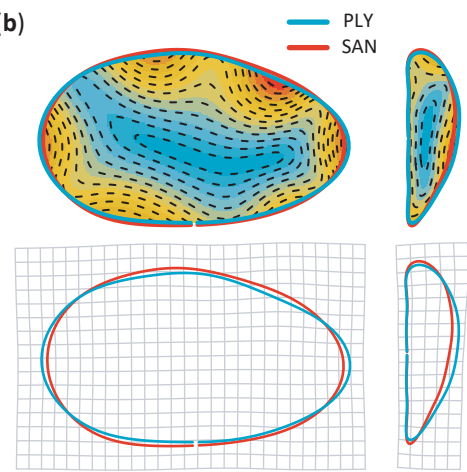

Supplement: Supplementary file 1 [file genes-11-00298-s001.zip › Supplementary_Material/SF_6.pdf]
